# SKIN NODULES AS A PRESENTING FEATURE OF DIFFUSE LARGE B-CELL GASTRIC LYMPHOMA

**DOI:** 10.4103/0019-5154.39744

**Published:** 2008

**Authors:** V Satya Suresh Attili, Ullas Batra, P P Bapsy, D Lokanatha, R Clementeena, P P Varma, M Malati, Kamal V S Saini

**Affiliations:** *From Department of Medical Oncology, Kidwai Memorial Institute of Oncology, Bangalore - 560 029, India. E-mail: sureshattili@yahoo.com*; 1 *From Department of Pathology, Kidwai Memorial Institute of Oncology, Bangalore - 560 029, India*; 2 *From Department of Surgical Oncology, Kidwai Memorial Institute of Oncology, Bangalore - 560 029, India*

Non-Hodgkin's lymphomas (NHLs) are a group of malignant disorders that can arise from any part of the lymphatic system. However, extra-nodal lymphomas are uncommon accounting for around 6-18% of NHLs. Among extra-nodal involvement, the gastrointestinal (GI) tract is the commonest system in around one out of three cases, the majority of which are low-grade MALT (Mucosa Associated Lymphoid Tumors). Stomach is the commonest organ with 50-75% of all GI NHL arising from the stomach; however, diffuse large B-cell variants are uncommon.^1^

We report a case of high-grade, diffuse large B-cell lymphoma of stomach in a 35-year-old female with disseminated skin involvement at presentation.

**Fig. 1:**
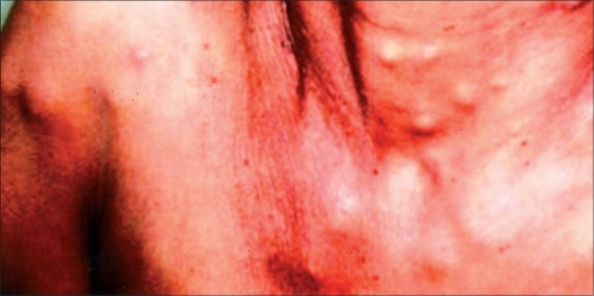
Skin nodules

**Fig. 2:**
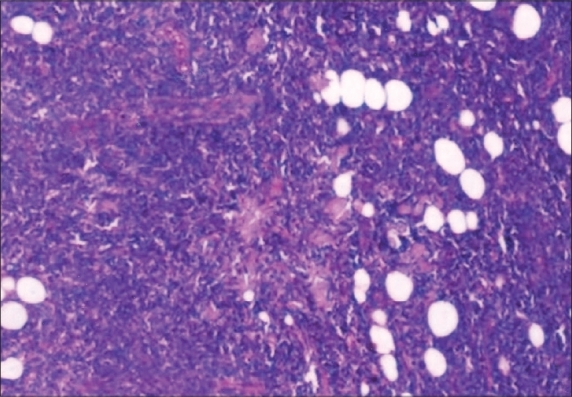
Histopathology of skin lesion

The patient presented with history of abdominal pain and multiple swellings over the neck and upper limbs for two months. Examination showed multiple subcutaneous nodules over the face, neck, shoulder, thorax and abdomen (Fig. 1) and the systemic examination was normal. Investigations revealed normal hematological and biochemical parameters. The CT scan of the abdomen revealed thickening of the stomach wall with perigastric lymphadenopathy. Upper gastrointestinal endoscopy revealed a small ulcerated lesion on the lesser curvature with multiple satellite lesions in the distal body. Biopsy from the lesion showed diffuse dense infiltration of lamina propria by small round cells. Fine needle aspiration cytology (FNAC) from the skin lesions also showed similar morphology (Fig. 2). Immunohistochemistry showed (done on cell blocks of FNAC from skin lesion and biopsy from stomach) similar results i.e. CK negative, LCA positive, CD 20 positive. Other staging workup including CT thorax and bone marrow examination were normal. As the current evidence suggests no role of surgery in Stage IV gastric lymphoma, the patient was offered combination chemotherapy with Cyclophosphamide 750 mg/m^2^, Doxorubicin 50 mg/m^2^ and Vincristine 1.4 mg/m^2^ each on Day 1, plus Prednisolone 100 mg on Days 1 to 5. She achieved major partial remission at the end of four cycles for the stomach and skin lesions.

The differential diagnosis in the present case includes disseminated lymphoma, (Stage IV) with stomach and skin involvement. However, as per the definition of primary gastric lymphoma, the patient had a primary lesion in the stomach, without systemic/peripheral adenopathy.^1^ Therefore in the present case the primary diagnosis is very clear. However, another possibility of primary cutaneous lymphoma with metastasis to stomach could also be thought of. Nevertheless we know that “malignant cells in cutaneous lymphomas express many surface molecules like CD45R0, a marker of ‘activated/memory’ T cells. They use surface proteins such as the cutaneous lymphoid antigen (CLA) and chemokine receptors to home to skin^2^”. The reported incidence of B-cell malignancies is extremely rare at <1% of all cutaneous lymphomas. Therefore, in the present case, as the patient had diffuse large B-cell lymphoma, the second possibility can be ruled out with reasonable confidence. In contrast to the predominance of the high-grade histological subtype in the rest of the GI tract, low-grade lymphomas are more commonly seen in the stomach, with the MALT subtype representing a majority of cases.^3^ Contrary to other low-grade nodal lymphomas, which usually present in the advanced stage, gastric lymphoma usually presents as a localized disease with only a minority of patients presenting in Stage IV.^3^ The commonest secondary site involved is bone marrow. MEDLAR search showed only two cases; one by Koch *et al.*^4^ and the other by Fischbach *et al.*^5^ (*n* = 266). To the best of our knowledge, it is the third reported case. The outcome is not defined in this subset. However, as the current evidence suggests that there is only minimal role of surgery and the mainstay of therapy is CHOP/CHOP-like regimens, the patient was offered CHOP and she showed a good response to therapy. The current case report reconfirms that chemotherapy without surgery can achieve good results even in advanced high-grade gastric lymphomas.
